# β-Bi_2_O_3_ Nanosheets Functionalized with Bisphenol A Synthetic Receptors: A Novel Material for Sensitive Photoelectrochemical Platform Construction

**DOI:** 10.3390/nano13050915

**Published:** 2023-03-01

**Authors:** Jing-Jing Yang, Ying-Zhuo Shen, Zheng Wang, Bo Zhou, Xiao-Ya Hu, Qin Xu

**Affiliations:** School of Chemistry and Chemical Engineering, Yangzhou University, Yangzhou 225002, China

**Keywords:** β-Bi_2_O_3_, nanosheet, PEC sensor, bisphenol A, molecularly imprinted

## Abstract

In this study, β-Bi_2_O_3_ nanosheets functionalized with bisphenol A (BPA) synthetic receptors were developed by a simple molecular imprinting technology and applied as the photoelectric active material for the construction of a BPA photoelectrochemical (PEC) sensor. BPA was anchored on the surface of β-Bi_2_O_3_ nanosheets via the self-polymerization of dopamine monomer in the presence of a BPA template. After the elution of BPA, the BPA molecular imprinted polymer (BPA synthetic receptors)-functionalized β-Bi_2_O_3_ nanosheets (MIP/β-Bi_2_O_3_) were obtained. Scanning electron microscopy (SEM) of MIP/β-Bi_2_O_3_ revealed that the surface of β-Bi_2_O_3_ nanosheets was covered with spherical particles, indicating the successful polymerization of the BPA imprinted layer. Under the best experimental conditions, the PEC sensor response was linearly proportional to the logarithm of BPA concentration in the range of 1.0 nM to 1.0 μM, and the detection limit was 0.179 nM. The method had high stability and good repeatability, and could be applied to the determination of BPA in standard water samples.

## 1. Introduction

Photoelectrochemical sensors (PECs) have attracted a great deal of attention due to their great potential in trace detection, and suitable semiconductor materials are the key to constructing PECs. As an emerging semiconductor material, Bi-based semiconductor materials have shown promise thanks to their advantages of easy fabrication, low price, non-toxicity, good environmental compatibility and good visible light response [1]. Due to the better stability of Bi^3+^ (the 6s orbital has an inert electron pair) and stronger visible light response (hybridized O 2p and Bi 6s orbitals shift the valence band (VB) up), most studies have focused on Bi^3+^-containing compounds such as Bi_2_O_3_ [2], BiVO_4_ [3], Bi_2_O_2_CO_3_ [4], Bi_2_WO_6_ [5], BiPO_4_ [6], BiFeO_3_ [7], BIOX (X=Cl, Br, I) [8,9,10] etc. Among them, as a p-type semiconductor, Bi_2_O_3_ has good biocompatibility, high stability and narrow band gap. These characteristics make Bi_2_O_3_ an extremely promising material in the construction of optoelectronic devices [11].

Generally, Bi_2_O_3_ exists in a total of six crystalline forms, including α-(monoclinic phase), β-(tetragonal phase), γ-(body-centered cubic phase), δ-(face-centered cubic phase), ε-(rhombohedral phase) and ω-(triclinic phase) [12]. Among these crystalline phases, β-Bi_2_O_3_, which is a metastable phase, has the strongest light absorption and a narrow band gap (2.19 eV), making β-Bi_2_O_3_ a strong candidate for photoelectrode materials [13,14,15,16]. Currently, most of the synthesis methods for β-Bi_2_O_3_ involve high temperature and pressure or complicated sample processing [17,18], which is incompatible with the concept of green chemistry. Here, a low-temperature CTAB-induced aqueous-phase crystallization method was applied for the synthesis of β-Bi_2_O_3_ [13]. Not only is the synthesis process simple and low-cost, but the synthesized β-Bi_2_O_3_ also has a stable photocurrent, which is of great benefit to the construction of PECs. Although β-Bi_2_O_3_ has been widely applied in photocatalysis [19,20], few applications of β-Bi_2_O_3_ in PECs construction have been reported [13].

Selectivity is another important factor that should be considered for the construction of PECs. In order to improve the specific recognition ability of PECs, some recognition elements, such as antibodies [21], enzymes [22], DNA aptamers [23] etc., are often introduced to combine with photoactive materials to form the sensor. In addition to such elements, molecular imprinted polymers (MIPs) have been regarded as promising synthetic receptors in the field of sensors due to their remarkable specific recognition function [24]. The synthesis strategy of MIPs is usually based on the formation of polymer in solvents between functional monomers and cross-linking agents, in which template molecules are embedded by covalent or non-covalent interactions (usually hydrogen bonds or van der Waals force [25]). The relationship between template molecules and imprinted polymer is similar to that between antigens and antibodies [26]. The obtained MIP generally has a complex and stable three-dimensional (3D) porous structure that matches with the target molecules. Thus, MIPs have been applied widely in different analytical methods owing to the advantages of recognition specificity and estimable structure [27]. It is very important to choose a suitable functional monomer for the preparation of MIPs. Dopamine can be self-polymerized to form polydopamine (PDA) films under weakly alkaline conditions (pH > 7.5) at room temperature. The active groups provided in the structure of PDA films include catechol, amine and imine, which are easily connected to molecules with hydroxyl structure by hydrogen bonding, and can achieve the in situ nucleation and growth of films with three-dimensional MIP cavity complementary to the target without additional reagents [28]. In recent years, MIPs derived from PDA have gradually seen use in sensor construction [29].

Bisphenol A (BPA), as one of the main substrates for the production of epoxy polyester resins and polycarbonate plastics, has been widely used in many consumer products in daily production and life [30]. However, its toxicity and estrogenic activity can interfere with the reproductive and endocrine systems of humans and wildlife [31]. Therefore, there is a need to develop a convenient and simple method for the quantitative analysis of BPA. Compared to the commonly used fluorescence [32], chromatography [33] and electrochemical methods [34], the PEC method has the advantages of low background current, inexpensive instrumentation, simple operation and fast response [35,36,37,38].

**Scheme 1:**
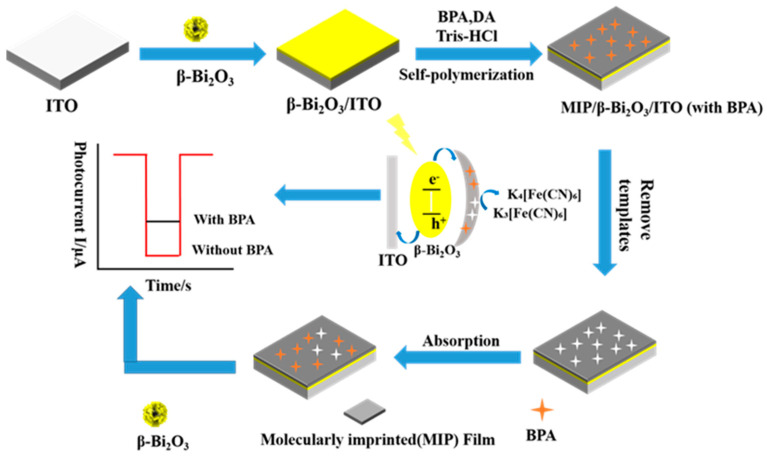
Schematic illustration of the β-Bi_2_O_3_-based MIP-PEC sensor for BPA detection.

In this work, a novel PEC sensor for BPA detection was constructed based on β-Bi_2_O_3_. Scheme 1 illustrates the construction process and detection mechanism of the PEC sensor. First, β-Bi_2_O_3_ was immobilized on an ITO surface as a PEC substrate to obtain the photocurrent signal. Then, MIP was formed on the surface of β-Bi_2_O_3_ by eluting the BPA template after the self-polymerization of DA in Tris-HCl. When the MIP-PEC sensor was irradiated by visible light, holes were transferred to the ITO electrode to generate photocurrent, and photogenerated electrons combined with the hole donor of K_3_;[Fe(CN)_6_] in the electrolyte to generate an amplified photocurrent signal which would improve the sensitivity. After the imprinted cavity was occupied by the template molecule BPA, fewer electron acceptors and less visible light could reach the electrode surface, yielding a reduced photocurrent for MIP/β-Bi_2_O_3_/ITO. The principle of BPA detection is based on the specific recognition ability of MIP. To our best knowledge, the application of β-Bi_2_O_3_ in MIP-PEC has not been reported. The method applied herein exhibited excellent performance in the detection of BPA in real samples.

## 2. Experimental

### 2.1. Reagents and Apparatus

The indium tin oxide coated glass (ITO, 8 Ω per square) was purchased from Zhuhai Kaivo Optoelectronic Technology Co., Ltd. (Zhuhai, China). Acetonitrile (ACN), acetic acid (HAC), ethanol (EtOH), tris (hydroxymethyl) aminomethane (Tris), potassium iodide (KI), bismuth nitrate pentahydrate (Bi(NO_3_)_3_·5H_2_O), cetyltrimethylammonium bromide (CTAB), sodium sulfate (Na_2_SO_4_), potassium ferricyanide (K_3_[Fe(CN)_6_]), potassium hexacyanoferrate (K_4_[Fe(CN)_6_]·3H_2_O), chitosan ((C_6_H_11_NO_4_)n), sodium hydroxide (NaOH), phenol (PH) and hydrogen chloride (HCl) were of analytical grade and purchased from Sinopharm Chemical Reagent Co., Ltd. (Shanghai, China). Bisphenol A (BPA), pyrocatechol (CT), resorcinol (INTER), p-dihydroxybenzene (HQ), 2,4-dichlorophenol (2,4-DCP) and dopamine (DA) were bought from Aladdin Biochemical Technology Co., Ltd. (Shanghai, China).

All electrochemical measurements were performed on a CHI660E electrochemical workstation (Chenhua Instrument Co., Ltd., Shanghai, China). The PEC experiments were performed on a CIMPS-2 workstation (Zennium, Zahner-Elektrik GmbH & CoKG, Germany). A 500 W WLC02 ((4300k) # 1522, Zahner-Elektrik) with a wavelength in the visible light spectrum was used as the irradiation source. A conventional three-electrode system was used which contained an Ag/AgCl (saturated KCl) electrode as the reference electrode. The morphologies of the samples were obtained on an S-4800 scanning electron microscope (SEM) (Hitachi Co., Ltd., Tokyo, Japan). Crystal structure analysis was performed with a Bruker D8 Advance X-ray diffractometer (XRD) (Bruker Scientific Technology Co., Ltd., Billerica, MA, USA) using Cu Kα radiation. The pH of solutions was adjusted using PHS-25 meter (Shanghai INESA Scientific Instrument Co., Ltd., Shanghai, China).

### 2.2. Synthesis of β-Bi_2_O_3_ Nanosheets

β-Bi_2_O_3_ nanosheets were obtained by a simple one-pot oil bath method [13]. Firstly, 0.5 mmol Bi(NO_3_)_3_·5H_2_O was added to 80 mL CTAB (4 mmol/L) solution with 0.08 mmol KI and dispersed uniformly by ultrasonic treatment. Then, the suspension was heated in an oil bath stirred by magnetic force at 353 K for 3 h. The precipitate was collected by washing with water and ethanol two times and dried in a vacuum oven at 333 K for 6 h. 

### 2.3. Fabrication of β-Bi_2_O_3_/ITO Electrode

Firstly, bare ITO glasses were ultrasonically cleaned with 0.1 M NaOH, surfactant, ethyl alcohol and deionized water before β-Bi_2_O_3_ modification. An amount of 5 mg of β-Bi_2_O_3_ was dispersed in 1 mL of 0.1% chitosan acetic acid solution by ultrasound. Then, 30 μL of the β-Bi_2_O_3_ ink was dropped onto the surface of the ITO and the modification area of the electrode was 1 cm^2^.

### 2.4. Functionalization of β-Bi_2_O_3_/ITO Electrode with BPA Synthetic Receptors

BPA (0.05 mmol) was dissolved in 1 mL acetonitrile solution, and then DA (10 mg) and 4 mL of Tris-HCl Buffer (10 mM pH = 8) were added into this solution. β-Bi_2_O_3_/ITO was placed in this solution, and self-polymerization was initiated at room temperature. Dopamine polymerization lasted for 40 min. After the reaction, the electrode was washed with deionized water and dried at room temperature. The electrode was then soaked in a solution consisting of 77 vol% water, 20 vol% acetonitrile and 3 vol% acetic acid (*v*/*v*/*v*, 77:20:3) [29] for 8 min to remove the imprinted BPA. As a control, the non-imprinted polymer NIP/β-Bi_2_O_3_/ITO was prepared by the above steps, without adding the template molecule BPA.

### 2.5. Detection of BPA

In this work, PEC measurements were carried out in a three-electrode system with MIP/β-Bi_2_O_3_/ITO or NIP/β-Bi_2_O_3_/ITO as the working electrode, Ag/AgCl as the reference electrode and platinum wire as the counter electrode. A 0.1 M Na_2_SO_4_ aqueous solution containing 10^–4^ M K_3_[Fe(CN)_6_] was used as the detection electrolyte. Due to the solubility limitation of BPA in water, mixed solutions of ethanol and water (*v*/*v*, 1:9) containing different concentrations of BPA were used as the incubation solutions. After being immersed in a static incubation solution for a period of time, the electrode was washed to remove the loosely bound BPA and put into the detection solution to check its response. All photocurrent measurements were performed at 0.0 V (vs. Ag/AgCl). The differences in the photocurrent of MIP/β-Bi_2_O_3_/ITO before and after the recognition of different concentrations of BPA were determined for the quantification of BPA.

## 3. Results and Discussion

### 3.1. Characterization of β-Bi_2_O_3_ and BPA Synthetic Receptors Functionalized β-Bi_2_O_3_

**Figure 1:**
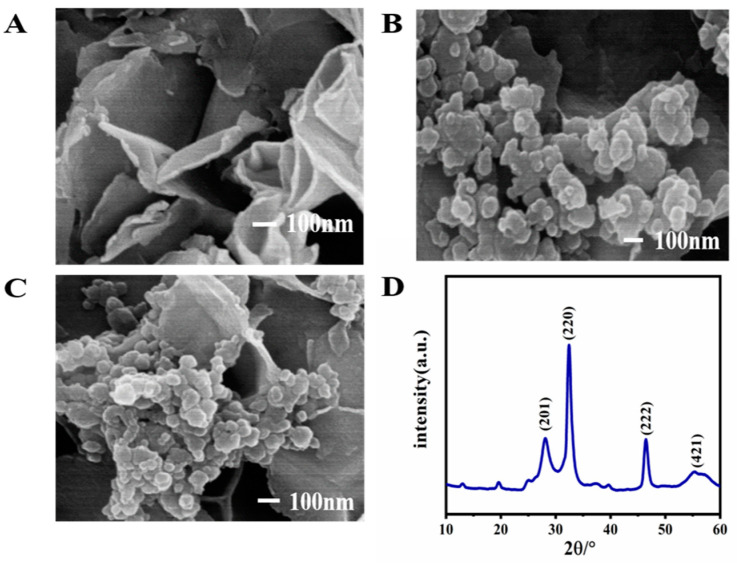
SEM images of (**A**) β-Bi_2_O_3_; (**B**) MIP/β-Bi_2_O_3_/ITO before elution and (**C**) after elution. (**D**) XRD pattern of β-Bi_2_O_3_.

SEM was used to evaluate the morphology of β-Bi_2_O_3_. As shown in Figure 1A, β-Bi_2_O_3_ showed a stacked petal-like two-dimensional sheet structure, which was consistent with the literature [13]. The existence of an internal electric field in the semiconductor with a layered structure enables both a significant separation of photogenerated charges and a significant increase in the efficiency of the use of photogenerated carriers [39].

The morphology of β-Bi_2_O_3_ functionalized with BPA synthetic receptors was also characterized by SEM. After molecular imprinting, some spherical particles were adhered to the lamellar β-Bi_2_O_3_ surface (Figure 1B), which indicated the successful polymerization of the BPA molecular layer. The surface morphology of the polymer did not differ much when the imprinted BPA molecules were removed by eluent treatment (Figure 1C). This means that the elution process did not destroy the structure of the imprint layer.

The crystalline phase of β-Bi_2_O_3_ was characterized by XRD (Figure 1D). For β-Bi_2_O_3_, all diffraction peaks matched well with the tetragonal crystal structure of β-Bi_2_O_3_ (JCPDS file 27-0050). The main diffraction peaks at 27.9°, 32.4°, 46.2° and 55.4° belonged to the (201), (220), (222) and (421) crystal planes of β-Bi_2_O_3_, respectively. In addition, no impurity peaks were observed in the X-ray diffraction peaks, indicating the high purity of the synthesized samples.

### 3.2. BPA Sensor Feasibility for BPA Detection

**Figure 2:**
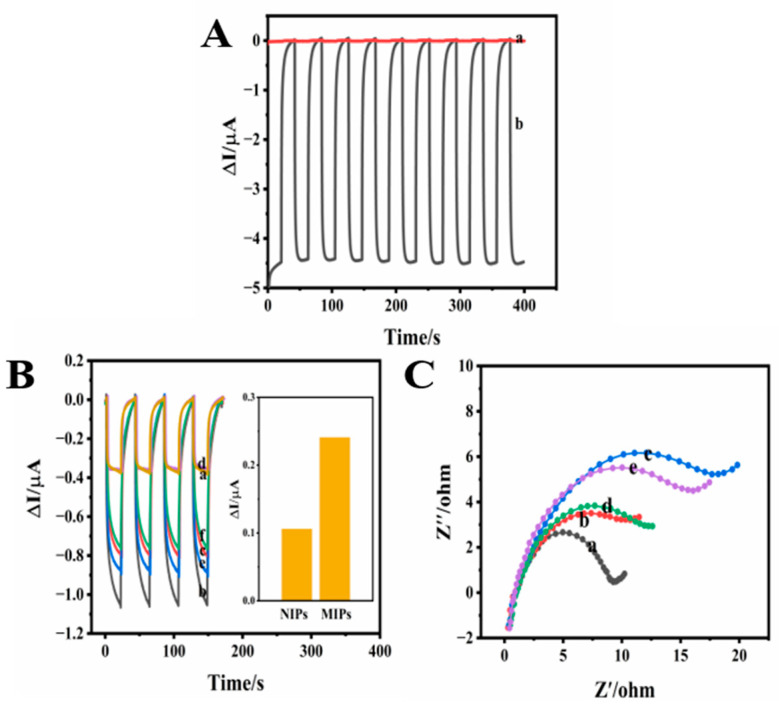
(**A**) The photocurrent comparison of β-Bi_2_O_3_ in 0.1 M Na_2_SO_4_ electrolyte under a light intensity of 500 W m^−2^ without (a) and with 10^−4^ M K_3_[Fe(CN)_6_] (b). (**B**) Photoelectrochemical behaviors of MIP and NIP. MIP/β-Bi_2_O_3_/ITO photocurrent responses after polymerization (a), after the elution of BPA (b), after the re-recognition of BPA (c); NIP/β-Bi_2_O_3_/ITO photocurrent responses after polymerization (d), after the elution step (e) and after the incubation with BPA (f). The concentration of BPA is 10 nM. (**C**) EIS characterization of ITO (a), β-Bi_2_O_3_/ITO (b), MIP/β-Bi_2_O_3_/ITO after polymerization (c), after the elution (d) and after the incubation of 10 nM BPA (e) in 5.0 mM [Fe(CN)_6_]^3−/4−^ solution containing 0.1 M KCl recorded in the frequency range from 0.1 Hz to 10 kHz with an open circuit potential.

After the construction of β-Bi_2_O_3_/ITO, we performed a series of electrochemical measurements to test the performance of the substrate. Figure 2A shows that the photocurrent of β-Bi_2_O_3_/ITO in pure Na_2_SO_4_ solution was small due to the rapid recombination of photogenerated electron–hole pairs in β-Bi_2_O_3_ [40], and the photocurrent signal increased dramatically by a factor of 100 after the addition of K_3_[Fe(CN)]_6_. As an electron scavenger, Fe^3+^ combined with the photogenerated electrons. This led to the suppression of the recombination of electron–hole pairs in β-Bi_2_O_3_, and the photocurrent increased sharply.

In order to evaluate the selective performance of MIP for BPA, photocurrent tests of different electrodes were performed. In Figure 2B, curve a represents the photocurrent of MIP/β-Bi_2_O_3_/ITO after self-polymerization. The photocurrent of MIP/β-Bi_2_O_3_/ITO was very weak because of the dense polymer film formed on the surface of β-Bi_2_O_3_. The photocurrent of NIP/β-Bi_2_O_3_/ITO after self-polymerization also supported this conclusion. After the elution, the photocurrent of the MIP-modified electrode increased sharply (curve b). Many cavities appeared after the elution, which reduced the mass-transfer resistance. After incubation in the BPA solution, the photocurrent decreased again due to the mass-transfer resistance of the adsorbed BPA (curve c). Curve e shows the values of photocurrent changes after NIP elution; the photocurrent increased as a result of the damage of the NIP by the eluent. Curve f shows the values of photocurrent changes after NIP adsorption, which were due to the non-specific adsorption of BPA by the hydroxyl groups on the NIP surface. The results show that the difference in photocurrent before and after MIP adsorption was 0.241 µA, which is 2.27 times higher than the photocurrent difference of NIP (0.106 µA). 

Electrochemical impedance spectroscopy (EIS) was further used to monitor the formation of PEC sensors, and to assess the electron transfer capacity at different electrode interfaces. The detection was performed in a 5 mM [Fe(CN)_6_]^3−/4−^ solution containing 0.1 M KCl, and the results are shown in Figure 2C. The bare ITO electrode (curve a) showed a low electron-transfer resistance due to its excellent conductivity. After modification of the β-Bi_2_O_3_ film on the ITO electrode (curve b), the electron-transfer resistance increased slightly due to the weak conductivity of the semiconductor. After polymerization (curve c), a dense non-conductive polymer was formed on the electrode surface, which increased the mass-transfer resistance, and the impedance value reached i maximum. When the template molecule was removed (curve d), an imprinted cavity was left on the composite surface, which reduced the impedance. However, when the template molecule BPA was reabsorbed, the imprinted cavity was occupied again, and the impedance increased (curve e). These results indicate the successful preparation of the MIP/β-Bi_2_O_3_/ITO sensor.

### 3.3. Optimization of the Experimental Conditions

#### 3.3.1. Optimization of MIP/β-Bi_2_O_3_/ITO Construction Conditions

**Figure 3:**
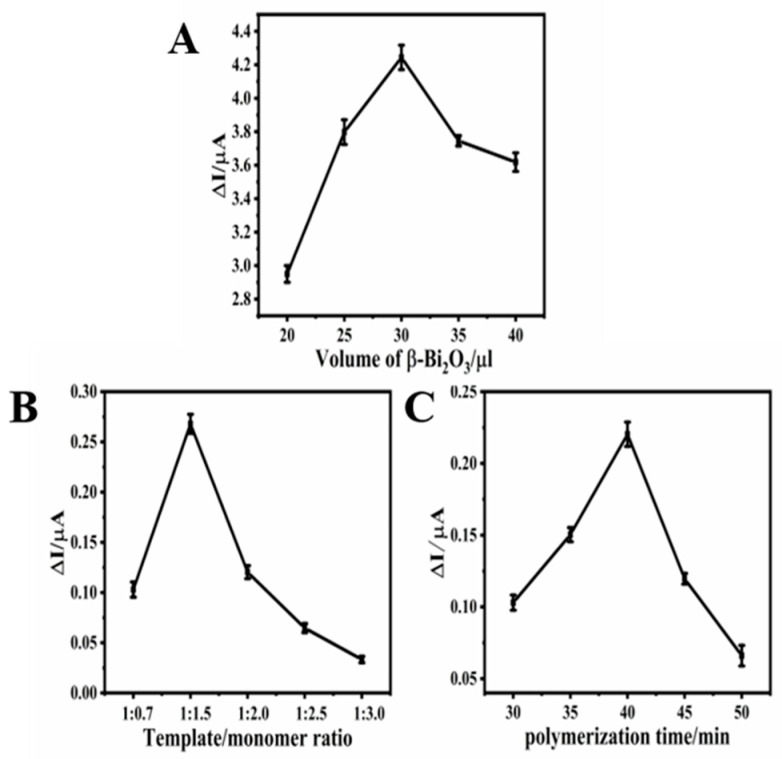
Optimization of (**A**) modification volume of β-Bi_2_O_3_; (**B**) template/monomer ratio. (**C**) self-polymerization time. The concentration of BPA is 10 nM.

The parameters of sensor construction were optimized. The modification amount of β-Bi_2_O_3_ was adjusted from 20 µL to 40 µL (Figure 3A). When the modification amount was 30 µL, the photocurrent response reached the maximum value. If the amount of material continued to increase, the material on the surface became too thick, and the holes could not reach the ITO surface quickly. Recombination quenching occurred, and it eventually led to poor photoresponse. In general, the ratio of template molecules to functional monomers affected the number of imprinted cavities in the MIP framework, which in turn affected the detection capability of the sensor. As shown in Figure 3B, as the content of functional monomers increased, the change of photocurrent increased, indicating that the ability to recognize BPA was gradually enhanced. It reached the maximum at 1:1.5. At this point, the MIP/β-Bi_2_O_3_/ITO produced enough binding sites, lending the ability to recognize the maximum BPA. When the amount of functional monomer continued to increase, the detection signal became weaker because the excess DA prevented the formation of effective hydrogen bonds with BPA. Therefore, the optimal molar ratio of BPA to DA was found to be 1:1.5. Self-polymerization time is another important parameter affecting the performance of MIP/β-Bi_2_O_3_/ITO electrodes. The thickness of the PDA layer grown on the electrode surface increased with the prolongation of polymerization time. As shown in Figure 3C, when the self-polymerization time increased, the photocurrent difference gradually increased, indicating that the performance of the sensor was improving. When the self-polymerization time reached 40 min, the photocurrent difference reached its maximum value. With increasing polymerization time, the current change decreased sharply. When the polymerization time was short, the PDA layer was too thin to form enough target sites. However, when the polymerization time exceeded 40 min, the PDA layer was too thick, and the BPA molecules embedded in the bottom layer were difficult to elute. On the basis of these results, the following experimental conditions were considered to be the best: (A) β-Bi_2_O_3_ modification volume, 30 µL; (B) ratio of template molecules to functional monomers, 1:1.5; (C) self-polymerization time, 40 min.

#### 3.3.2. Optimization of MIP/β-Bi_2_O_3_/ITO Detection Conditions

**Figure 4:**
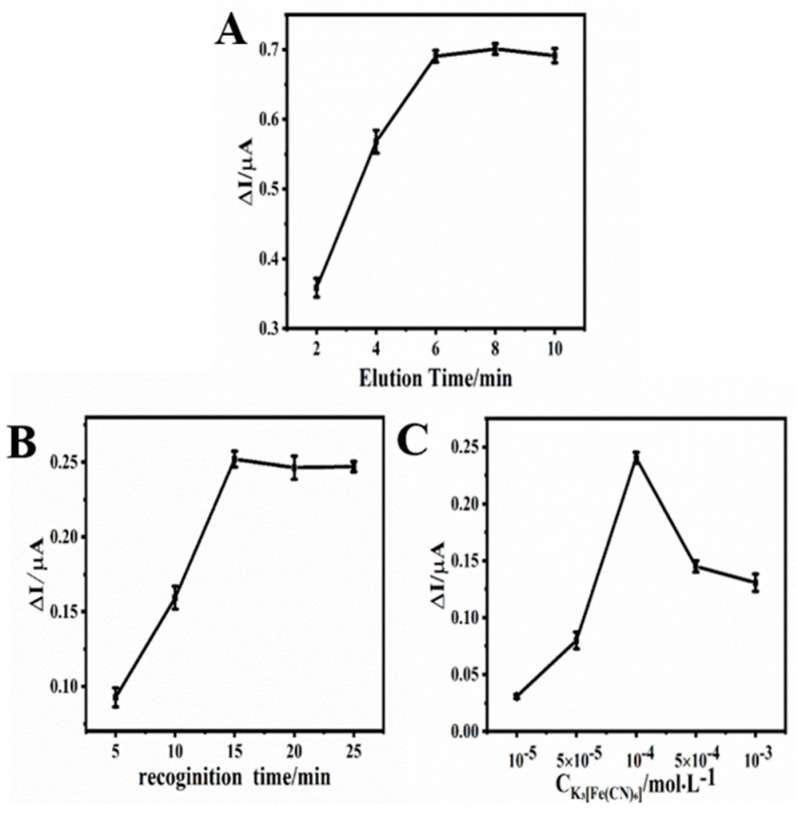
Optimization of (**A**) elution time; (**B**) recognition time; (**C**) concentration of K_3_[Fe(CN)_6_]. The concentration of BPA is 10 nM.

After the construction of the MIP/β-Bi_2_O_3_/ITO, the elution time, recognition time and concentration of K_3_[Fe(CN)_6_] were optimized to obtain the best sensor performance. The elution of template molecules would affect the number of BPA cavities blotted in the MIP. In this experiment, 20 vol% acetonitrile and 3 vol% acetic acid aqueous solution was used as eluent to remove the template molecule. As shown in Figure 4A, as the elution time increased, the current of the electrode increased gradually. When the elution time reached 8 min, the current reached a plateau. Even if the elution time was longer, the current was almost unchanged, which indicated that the BPA molecules had been completely removed. Therefore, 8 min was chosen as the optimal elution time in this work. After the template molecules were eluted from the polymer membrane, the MIP/β-Bi_2_O_3_/ITO was soaked in the recognition solution (a mixed solution of ethanol and water (1:9) containing 10 nM BPA) for different lengths of time. Figure 4B shows that the current increased gradually and reached a maximum at 15 min. After 15 min, the absorption of BPA reached saturation, and the current did not change with increasing recognition time. Therefore, 15 min was chosen as the best recognition time. K_3_[Fe(CN)_6_] was selected as the signal amplification unit in this experiment. The sensor was measured in different concentrations of K_3_[Fe(CN)_6_] to study its concentration effect on the sensor performance. As shown in Figure 4C, when the K_3_[Fe(CN)_6_] concentration was increased from 10^−5^ to 10^−4^ M, the current gradually increased, and reached its maximum at 10^−4^ M K_3_[Fe(CN)_6_]. When the concentration of K_3_[Fe(CN)_6_] was below 10^−4^ M, the amount of Fe^3+^ that could reach the β-Bi_2_O_3_ layer was small, and the inhibition effect on the recombination of electrons and holes was poor. With further increase of K_3_[Fe(CN)_6_] concentration, the current began to decrease. As the concentration of K_3_[Fe(CN)_6_] was too high, it impeded the hole transfer, having a negative effect on the separation of photogenerated electron–hole pairs [41]. Therefore, the detection conditions were optimized to be the best: (A) elution time, 8 min; (B) recognition time, 15 min; (C) concentration of K_3_[Fe(CN)_6_], 10^−4^ M.

### 3.4. Evaluation of the Performance of the β-Bi_2_O_3_-Based MIP-PEC Sensor

**Figure 5:**
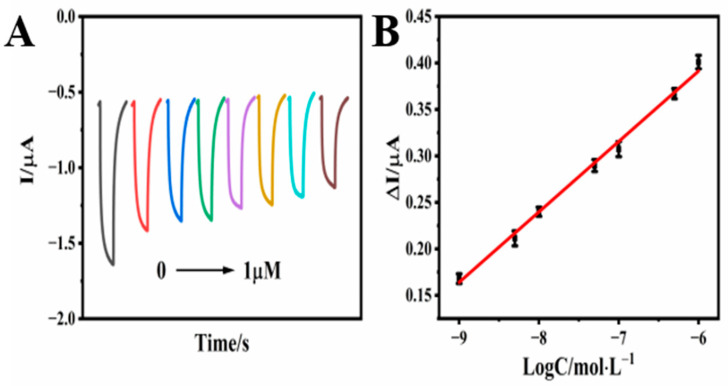
(**A**) Photocurrents of the MIP/β-Bi_2_O_3_/ITO PECs to different concentrations of BPA (0, 1 nM, 5 nM, 10 nM, 50 nM, 100 nM, 0.5 μM and 1 μM). (**B**) Linear calibration curves for MIP/β-Bi_2_O_3_/ITO (error bars indicate the standard deviation (n = 3)).

Under optimal experimental conditions, the photocurrent responses of the MIP/β-Bi_2_O_3_/ITO sensor to different concentrations of BPA were analyzed. Figure 5A shows that the photocurrent decreased with increasing BPA concentration. In the range of 1 nM–1 μM, the photocurrent was linearly related to the logarithm of the BPA concentration. The linear equation was ΔI (μA) = 0.84576 + 0.07575 logC (mol/L) (R^2^ = 0.9955). The detection limit obtained by the determination was 0.179 nM (calculation formula is 3σ/k, where σ is the standard deviation of the intercept and k is the slope of the calibration curve [42]) and the sensor sensitivity was 0.07575 µA (logM)^−1^ cm^−2^. The MIP/β-Bi_2_O_3_/ITO exhibited better sensing performance in terms of wider linear range and lower detection limit compared to some other relevant reported BPA detection methods (Table 1), and could be used for the detection of BPA in real samples. 

### 3.5. Stability, Reproducibility and Selectivity of the β-Bi_2_O_3_-Based MIP-PEC Sensor

**Figure 6:**
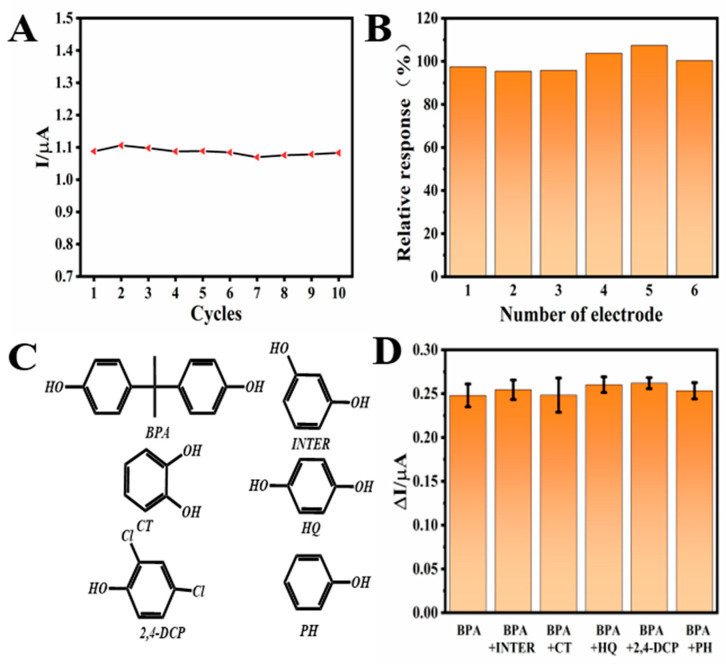
(**A**) Stability of β-Bi_2_O_3_-based MIP-PEC sensor. (**B**) Reproducibility of the β-Bi_2_O_3_-based MIP-PEC sensor. (**C**) The structural formulas of the interferents. (**D**) Selectivity of MIP/β-Bi_2_O_3_/ITO to BPA and interferents. The concentration of BPA was 10 nM, and the interferents were at a concentration of 100 nM.

For further evaluation, the sensor was tested for stability, reproducibility and selectivity. As shown in Figure 6A, the photocurrent measurement of the sensor under 10 light-on tests could be seen under a continuous test of 400 s. The photocurrent response of the sensor maintained 99.56% of the original value (RSD = 1.16%), indicating that the sensor has excellent stability. To further examine the storage stability, the sensors were kept in a sealed glass bottle at 4 ℃ for two weeks. The data showed that the sensor maintained 96.84% of its initial response value after the two-week storage, indicating that the sensor has good storage stability. In terms of reproducibility measurement, the six independently produced electrodes were tested, and the resulting RSD was 4.36%, which also proved that the sensor has good reproducibility (Figure 6B). To test the selectivity of the sensor, structural analogues such as resorcinol (INTER), pyrocatechol (CT), p-dihydroxybenzene (HQ), 2,4-dichlorophenol (2,4-DCP) and phenol (PH) were selected as co-existing interferers (Figure 6C). Figure 6D shows that in the presence of 100 nM interferent, there was no significant change in 10 nM BPA detection. This indicates that the β-Bi_2_O_3_-based MIP-PEC sensor has a specific recognition ability for the detection of BPA. 

### 3.6. Real Sample Analysis

In order to verify the practicability of the MIP/β-Bi_2_O_3_/ITO sensor, we chose bottled water as a real sample, and tested the recovery rate by adding BPA. As shown in Table 2, bottled water itself did not contain BPA. Different concentrations of BPA (1 nM, 10 nM, 20 nM, 40 nM, 80 nM) were added to the samples, and the recovery tests showed that the recovery rates ranged from 97.3% to 103.5%. The recovery tests verified the feasibility of the PEC sensor for real water sample analysis.

## 4. Conclusions

In this work, β-Bi_2_O_3_ nanosheets were synthesized by a low-temperature, one-step, aqueous-phase crystallization method. The obtained nanosheet was stable and had good visible light response. Furthermore, BPA synthetic receptors were anchored on the β-Bi_2_O_3_ nanosheets by a simple self-polymerization process. The β-Bi_2_O_3_ functionalized with BPA synthetic receptors was further applied for BPA detection. The whole construction process is green and safe. The as-obtained sensor provides a fast, convenient and effective analytical method for the detection of BPA with good selectivity, repeatability and stability. The method performed well in actual sample detection, and has good application prospects in the field of environmental detection. The results also demonstrate that β-Bi_2_O_3_ is a promising substrate material with high stability and strong visible light absorption for PEC sensors. In the future, there will be more possibilities for β-Bi_2_O_3_ to be used in PECs construction.

## Figures and Tables

**Table 1:** Comparison of different BPA detection methods.

Method	Liner Range (μM)	LOD (nM)	Ref.
MIP@CDs(FD ^a^)	0.1–4.2	30	[43]
NPG(ED ^b^)	0.1–50	12.1	[44]
β-CD/ILCPE(SILs-SPE-ED ^c^)	0.04–1	4.16	[45]
Graphene/Ag/Si(GSPME-SERS ^d^)	0.00877–0.439	4.39	[46]
LLE-GC-MS ^e^	0.088–2.2	26.3	[47]
MIP/β-Bi_2_O_3_/ITO(PEC)	0.001–1	0.179	This work
NIP/β-Bi_2_O_3_/ITO(PEC)	—	4.397	This work

^a^ FD: fluorescence detection. ^b^ ED: electrochemical detection. ^c^ SILs-SPE-ED: supported ionic liquids solid-phase extraction coupled to electrochemical detection. ^d^ GSPME-SERS: solid-phase microextraction-surface enhanced Raman spectroscopy. ^e^ LLE-GC-MS: liquid–liquid extraction-gas chromatography–mass spectrometry.

**Table 2:** Detection of BPA added in bottled water.

Sample	Added (nM)	Found (nM)	Recovery (%)	RSD (%)
1	0.00	nd	—	—
2	1.00	1.04	103.5	2.47
2	10.00	10.13	101.3	2.30
3	20.00	20.51	102.6	1.85
4	40.00	39.39	98.2	2.22
5	80.00	77.88	97.3	1.44

## Data Availability

Not applicable.

## References

[1] Yu S.-Y., Zhang L., Zhu L.-B., Gao Y., Fan G.-C., Han D.M., Chen G., Zhao W.-W. (2019). Bismuth-containing semiconductors for photoelectrochemical sensing and biosensing. Coord. Chem. Rev..

[2] Yin S., Shao Y., Hu Q., Chen Y., Ding P., Xia J., Li H. (2021). In situ preparation of Bi_2_O_3_/(BiO)(_2_)CO_3_ composite photocatalyst with enhanced visible-light photocatalytic activity. Res. Chem. Intermed..

[3] Jin S., Ma X., Pan J., Zhu C., Saji S.E., Hu J., Xu X., Sun L., Yin Z. (2021). Oxygen vacancies activating surface reactivity to favor charge separation and transfer in nanoporous BiVO_4_ photoanodes. Appl. Catal. B-Environ..

[4] Zhang Q., Xu B., Yuan S., Zhang M., Ohno T. (2017). Improving g-C_3_N_4_ photocatalytic performance by hybridizing with Bi_2_O_2_CO_3_ nanosheets. Catal. Today.

[5] Zhou Y., Lv P., Meng X., Tang Y., Huang P., Chen X., Shen X., Zeng X. (2017). CTAB-Assisted Fabrication of Bi_2_WO_6_ Thin Nanoplates with High Adsorption and Enhanced Visible Light-Driven Photocatalytic Performance. Molecules.

[6] Lu M., Yuan G., Wang Z., Wang Y., Guo J. (2015). Synthesis of BiPO_4_/Bi_2_S_3_ Heterojunction with Enhanced Photocatalytic Activity under Visible-Light Irradiation. Nanoscale Res. Lett..

[7] Lu H., Du Z., Wang J., Liu Y. (2015). Enhanced photocatalytic performance of Ag-decorated BiFeO_3_ in visible light region. J. Sol-Gel Sci. Technol..

[8] Wang Q., Hui J., Li J., Cai Y., Yin S., Wang F., Su B. (2013). Photodegradation of methyl orange with PANI-modified BiOCl photocatalyst under visible light irradiation. Appl. Surf. Sci..

[9] Liu Q.Y., Han G., Zheng Y.F., Song X.C. (2018). Synthesis of BiOBrxI1-x solid solutions with dominant exposed {001} and {110} facets and their visible-light-induced photocatalytic properties. Sep. Purif. Technol..

[10] Hou J., Jiang K., Shen M., Wei R., Wu X., Idrees F., Cao C. (2017). Micro and nano hierachical structures of BiOI/activated carbon for efficient visible-light-photocatalytic reactions. Sci. Rep..

[11] Chen X., Wu W., Zhang Q., Wang C., Fan Y., Wu H., Zhang Z. (2022). Z-scheme Bi_2_O_3_/CuBi_2_O_4_ heterojunction enabled sensitive photoelectrochemical detection of aflatoxin B1 for health care, the environment, and food. Biosens. Bioelectron..

[12] Kanagaraj T., Kumar P.S.M., Thomas R., Kulandaivelu R., Subramani R., Mohamed R.N., Lee S., Chang S.W., Chung W.J., Nguyen D.D. (2022). Novel pure alpha-, beta-, and mixed-phase alpha/beta-Bi_2_O_3_ photocatalysts for enhanced organic dye degradation under both visible light and solar irradiation. Environ. Res..

[13] Wang H., Liang D., Xu Y., Liang X., Qiu X., Lin Z. (2021). A highly efficient photoelectrochemical sensor for detection of chlorpyrifos based on 2D/2D beta-Bi_2_O_3_/g-C_3_N_4_ heterojunctions. Environ. Sci. Nano.

[14] Praveen S., Veeralingam S., Badhulika S. (2021). A Flexible Self-Powered UV Photodetector and Optical UV Filter Based on beta-Bi_2_O_3_/SnO_2_ Quantum Dots Schottky Heterojunction. Adv. Mater. Interfaces.

[15] Park S., Ko H., Lee S., Kim H., Lee C. (2014). Light-activated gas sensing of Bi_2_O_3_-core/ZnO-shell nanobelt gas sensors. Thin Solid Film..

[16] Yang S., Jiao S., Nie Y., Lu H., Liu S., Zhao Y., Gao S., Wang D., Wang J., Li Y. (2022). A self-powered high performance UV-Vis-NIR broadband photodetector based on beta-Bi2O3 nanoparticles through defect engineering. J. Mater. Chem. C.

[17] Zahid A.H., Han Q., Jia X., Li S., Hangjia H., Liu H. (2020). Highly stable 3D multilayered nanoparticles-based beta-Bi_2_O_3_ hierarchitecture with enhanced photocatalytic activity. Opt. Mater..

[18] Shi Y., Luo L., Zhang Y., Chen Y., Wang S., Li L., Long Y., Jiang F. (2017). Synthesis and characterization of alpha/beta-Bi_2_O_3_ with enhanced photocatalytic activity for 17 alpha-ethynylestradiol. Ceram. Int..

[19] Guadalupe Yanez-Cruz M., Villanueva-Ibanez M., Mendez-Arriaga F., Alexander Lucho-Constantino C., de los Angeles Hernandez-Perez M., del Rocio Ramirez-Vargas M., Antonio Flores-Gonzalez M. (2022). Green route synthesis and characterization of beta-Bi_2_O_3_/SiO_2_ and beta-Bi_2_O_3_/Bi_2_O_2.75_/SiO_2_ using Juglans regia L. shell aqueous extract and photocatalytic properties for the degradation of RB-5. J. Anal. Sci. Technol..

[20] Xie T., Yang J., Penga Y., Wang J., Liu S., Xu L., Liu C. (2019). beta-Bi_2_O3/SrFe_12_O_19_ magnetic photocatalyst: Facile synthesis and its photocatalytic activity. Mater. Technol..

[21] Zang Y., Ju Y., Hu X., Zhou H., Yang Z., Jiang J., Xue H. (2018). WS_2_ nanosheets-sensitized CdS quantum dots heterostructure for photoelectrochemical immunoassay of alpha-fetoprotein coupled with enzyme-mediated biocatalytic precipitation. Analyst.

[22] Yang Z., Shi Y., Liao W., Yin H., Ai S. (2016). A novel signal-on photoelectrochemical biosensor for detection of 5-hydroxymethylcytosine based on in situ electron donor producing strategy and all wavelengths of light irradiation. Sens. Actuators B-Chem..

[23] Xu F., Zhu Y.-C., Ma Z.-Y., Zhao W.-W., Xu J.-J., Chen H.-Y. (2016). An ultrasensitive energy-transfer based photoelectrochemical protein biosensor. Chem. Commun..

[24] Yang X., Gao Y., Ji Z., Zhu L.B., Yang C., Zhao Y., Shu Y., Jin D., Xu Q., Zhao W.-W. (2019). Dual Functional Molecular Imprinted Polymer-Modified Organometal Lead Halide Perovskite: Synthesis and Application for Photoelectrochemical Sensing of Salicylic Acid. Anal. Chem..

[25] Cheong W.-J., Ali F., Choi J.-H., Lee J.-O., Sung K.-Y. (2013). Recent applications of molecular imprinted polymers for enantio-selective recognition. Talanta.

[26] Luan J., Xu T., Cashin J., Morrissey J.J., Kharasch E.D., Singamaneni S. (2018). Environmental Stability of Plasmonic Biosensors Based on Natural versus Artificial Antibody. Anal. Chem..

[27] Yang Y., Cao Y., Wang X., Fang G., Wang S. (2015). Prussian blue mediated amplification combined with signal enhancement of ordered mesoporous carbon for ultrasensitive and specific quantification of metolcarb by a three-dimensional molecularly imprinted electrochemical sensor. Biosens. Bioelectron..

[28] Nie Y., Liu Y., Su X., Ma Q. (2019). Nitrogen-rich quantum dots-based fluorescence molecularly imprinted paper strip for p-nitroaniline detection. Microchem. J..

[29] Miao J., Liu A., Wu L., Yu M., Wei W., Liu S. (2020). Magnetic ferroferric oxide and polydopamine molecularly imprinted polymer nanocomposites based electrochemical impedance sensor for the selective separation and sensitive determination of dichlorodiphenyltrichloroethane (DDT). Anal. Chim. Acta.

[30] Zwierello W., Maruszewska A., Skorka-Majewicz M., Goschorska M., Baranowska-Bosiacka I., Dec K., Styburski D., Nowakowska A., Gutowska I. (2020). The influence of polyphenols on metabolic disorders caused by compounds released from plastics—Review. Chemosphere.

[31] Tarafdar A., Sirohi R., Balakumaran P.A., Reshmy R., Madhavan A., Sindhu R., Binod P., Kumar Y., Kumar D., Sim S.J. (2022). The hazardous threat of Bisphenol A: Toxicity, detection and remediation. J. Hazard. Mater..

[32] Zhang L., Er J.C., Xu W., Qin X., Samanta A., Jana S., Lee C.-L.K., Chang Y.-T. (2014). “Orange alert”: A fluorescent detector for bisphenol A in water environments. Anal. Chim. Acta.

[33] Zhuang Y., Zhou M., Gu J., Li X. (2014). Spectrophotometric and high performance liquid chromatographic methods for sensitive determination of bisphenol A. Spectrochim. Acta Part A-Mol. Biomol. Spectrosc..

[34] Jang J., Kim D.-H., Lee W.-Y. (2016). Electrochemical Determination of Bisphenol A by Single-Walled Carbon Nanotube Composite Glassy Carbon Electrode. Anal. Lett..

[35] Jin D., Xu Q., Yu L., Hu X. (2015). Photoelectrochemical detection of the herbicide clethodim by using the modified metal-organic framework amino-MIL-125(Ti)/TiO_2_. Microchim. Acta.

[36] Wang J., Xu Q., Xia W.W., Shu Y., Jin D., Zang Y., Hu X. (2018). High sensitive visible light photoelectrochemical sensor based on in-situ prepared flexible Sn_3_O_4_ nanosheets and molecularly imprinted polymers. Sens. Actuators B-Chem..

[37] Cao Y., Wang L., Wang C., Hu X., Liu Y., Wang G. (2019). Sensitive detection of glyphosate based on a Cu-BTC MOF/g-C_3_N_4_ nanosheet photoelectrochemical sensor. Electrochim. Acta.

[38] Cao Y., Wang L., Wang C., Su D., Liu Y., Hu X. (2019). Photoelectrochemical determination of malathion by using CuO modified with a metal-organic framework of type Cu-BTC. Microchim. Acta.

[39] Wu X., Toe C.Y., Su C., Ng Y.H., Amal R., Scott J. (2020). Preparation of Bi-based photocatalysts in the form of powdered particles and thin films: A review. J. Mater. Chem. A.

[40] Zhang Z., Jiang D., Xing C., Chen L., Chen M., He M. (2015). Novel AgI-decorated beta-Bi_2_O_3_ nanosheet heterostructured Z-scheme photocatalysts for efficient degradation of organic pollutants with enhanced performance. Dalton Trans..

[41] Wang X., Deng H., Wang C., Wei Q., Wang Y., Xiong X., Li C., Li W. (2020). A pro-gastrin-releasing peptide imprinted photoelectrochemical sensor based on the in situ growth of gold nanoparticles on a MoS_2_ nanosheet surface. Analyst.

[42] Yu L., Zhang Q., Jin D., Xu Q., Hu X. (2019). A promising voltammetric biosensor based on glutamate dehydrogenase/Fe_3_O_4_/graphene/chitosan nanobiocomposite for sensitive ammonium determination in PM2.5. Talanta.

[43] Liu G., Chen Z., Jiang X., Feng D.-Q., Zhao J., Fan D., Wang W. (2016). In-situ hydrothermal synthesis of molecularly imprinted polymers coated carbon dots for fluorescent detection of bisphenol A. Sens. Actuators B-Chem..

[44] Yan X., Zhou C., Yan Y., Zhu Y. (2015). A Simple and Renewable Nanoporous Gold-based Electrochemical Sensor for Bisphenol A Detection. Electroanalysis.

[45] Huang L.-L., Huang Y., Chen Y.-K., Ding Y.-H., Zhang W.-F., Li X.-J., Wu X.-P. (2015). Supported Ionic Liquids Solid-Phase Extraction Coupled to Electrochemical Detection for Determination of Trace Bisphenol A. Chin. J. Anal. Chem..

[46] Liu Q., Zeng X., Liu Q., Hou X., Tian Y., Wu L. (2018). Sensitive detection of bisphenol A by coupling solid phase microextraction based on monolayer graphene-coated Ag nanoparticles on Si fibers to surface enhanced Raman spectroscopy. Talanta.

[47] Vilchez J.L., Zafra A., Gonzalez-Casado A., Hontorio E., del Olmo M. (2001). Determination of trace amounts of bisphenol F, bisphenol A and their diglycidyl ethers in wastewater by gas chromatography-mass spectrometry. Anal. Chim. Acta.

